# Human Macrophages Escape Inhibition of Major Histocompatibility Complex-Dependent Antigen Presentation by Cytomegalovirus and Drive Proliferation and Activation of Memory CD4^+^ and CD8^+^ T Cells

**DOI:** 10.3389/fimmu.2018.01129

**Published:** 2018-05-25

**Authors:** Giada Frascaroli, Carina Lecher, Stefania Varani, Corinna Setz, Johannes van der Merwe, Wolfram Brune, Thomas Mertens

**Affiliations:** ^1^Institute of Virology, Ulm University Medical Center, Ulm, Germany; ^2^Heinrich Pette Institute, Leibniz Institute for Experimental Virology, Hamburg, Germany; ^3^Department of Diagnostic, Experimental and Specialty Medicine, University of Bologna, Bologna, Italy; ^4^Institute of Molecular Virology, Ulm University Medical Center, Ulm, Germany

**Keywords:** human cytomegalovirus, macrophages, US2-11 immune evasive genes, major histocompatibility complex molecules, T-cell proliferation, T-cell activation

## Abstract

Human cytomegalovirus (HCMV) persistently infects 40–90% of the human population but in the face of a normal immune system, viral spread and dissemination are efficiently controlled thus preventing clinically signs and disease. HCMV-infected hosts produce a remarkably large amount of HCMV-specific CD4^+^ and CD8^+^ T cells that can even reach 20–50% of total T memory cells in the elderly. How HCMV may elicit such large and long-lasting T-cell responses in the absence of detectable viremia has not been elucidated yet. Additionally, HCMV is known to encode several gene products that potently inhibit T-cell recognition of infected cells. The best characterized are the four immune evasive US2, US3, US6, and US11 genes that by different mechanisms account for major histocompatibility complex (MHC) class I and class II degradation and intracellular retention in infected cells. By infecting M1 and M2 human macrophages (Mφ) with the wild-type HCMV strain TB40E or a mutant virus deleted of the four immune evasive genes US2, US3, US6, and US11, we demonstrated that human Mφ counteract the inhibitory potential of the US2-11 genes and remain capable to present peptides *via* MHC class I and class II molecules. Moreover, by sorting the infected and bystander cells, we provide evidence that both infected and bystander Mφ contribute to antigen presentation to CD4^+^ and CD8^+^ T cells. The T cells responding to TB40E-infected Mφ show markers of the T effector memory compartment, produce interferon-γ, and express the lytic granule marker CD107a on the cell surface, thus mirroring the HCMV-specific T cells present in healthy seropositive individuals. All together, our findings reveal that human Mφ escape inhibition of MHC-dependent antigen presentation by HCMV and continue to support T cell proliferation and activation after HCMV infection. Taking into account that Mφ are natural targets of HCMV infection and a site of viral reactivation from latency, our findings support the hypothesis that Mφ play crucial roles for the lifelong maintenance and expansion of HCMV-committed T cells in the human host.

## Introduction

Human cytomegalovirus (HCMV) is a complex DNA virus that belongs to the *Herpesviridae* family and infects a large proportion of the human population (infection rates range from 40 to 100% depending on the socioeconomic conditions). While in subjects with immature or deficient immune system HCMV is a serious cause of morbidity and mortality, in immunocompetent hosts the virus generally causes an asymptomatic and self-limiting primary infection followed by lifelong persistence (1). After primary HCMV infection, immunocompetent individuals produce neutralizing antibodies (2, 3) as well as high amounts of HCMV-specific T cells (4–6) that control viral replication and protect the host from HCMV disease [reviewed in Ref. (7)]. After resolution of the primary infection and throughout life, HCMV-specific T cells are maintained and expanded (8) reaching enormous amounts that dominate over any other chronic pathogen such as Epstein–Barr virus (EBV) and HIV (9). While in young healthy HCMV carriers an average of 10% of memory CD4^+^ and CD8^+^ T-cell pools is devoted to recognize HCMV peptides, in the elderly HCMV-specific T cells can reach up to 20–50% of the total T cells (10–12).

Such a large and sustained HCMV-specific T-cell response has been explained hypothesizing that clinically unapparent HCMV reactivations and low-grade local HCMV replication do take frequently place in the host and provide sufficient infected cells and viral antigens for a steady T-cell boost (13, 14). Two peculiar features of HCMV weaken this otherwise logic explanation. On the one hand, HCMV is a champion of immune modulation and encodes numerous proteins that can interfere with the host’s ability to efficiently recognize and clear virus-infected cells (15). On the other hand, HCMV has a unique capability to sabotage the most potent antigen-presenting cells (APC), namely, the dendritic cells (DC) [reviewed in Ref. (16)]. Among multiple HCMV immune evasive genes, four glycoproteins encoded by the unique short (US) region of the HCMV genome, i.e., US2, US3, US6, and US11, have been found to cause major histocompatibility complex (MHC) molecules downregulation and to prevent T-cell recognition of infected fibroblast [reviewed in Ref. (17–20)]. Moreover, it has been extensively reported that upon HCMV infection, DC undergo downregulation of MHC class I and class II molecules and become unable to efficiently stimulate T-cell responses (21, 22).

Since APC are necessary for the lifelong maintenance and expansion of HCMV-specific T cells, we hypothesized that other professional APC might play major roles in HCMV antigen presentation to T cells. Several lines of evidence support the hypothesis that macrophages (Mφ) may be able to escape HCMV immune evasion and play essential roles in anti-HCMV immune responses. First, it has already been shown in the mouse model of cytomegalovirus infection, that after MCMV infection primary Mφ resist MHC class I inhibition, present viral peptides, and stimulate cytotoxic T cells (23). Second, Mφ support persistent HCMV infection *in vitro* (24–26) as well as *in vivo* (27), and are a site of viral reactivation from latency (28, 29), thus representing a first site of production of viral antigens to be processed and presented to T cells. Finally, Mφ are long living and similarly to DC are equipped with the molecular machinery necessary for professional CD4^+^ and CD8^+^ T-cell stimulation including constitutive high levels of MHC molecules, expression of co-stimulatory molecules and secretion of soluble cytokines (30, 31).

In our previous study, we have shown that monocyte-derived pro-inflammatory M1-Mφ and anti-inflammatory M2-Mφ are susceptible to HCMV infection, secrete a plethora of pro-inflammatory cytokines and stimulate the proliferation of autologous T cells (25). In this study, we focused on the roles of the four US2-11 major determinants of MHC class I downregulation and we have compared the effects exerted by the wild-type endothelial cell tropic HCMV strain TB40E and its derivative mutant lacking the MHC downregulating genes US2-6 and US11 (RVTB40/E DeltaUS11). By applying new approaches for the differential analysis of HCMV-infected and bystander Mφ, based on flow cytofluorimetry double stainings as well as on the differential sorting of the infected and bystander Mφ, we demonstrated that the MHC downregulation mediated by pUS2-US11 is incomplete in Mφ and that HCMV-infected Mφ remain fully capable to stimulate the proliferation and antiviral activation of CD4^+^ and CD8^+^ T cells.

## Materials and Methods

### Ethic Statement

Buffy-coats were purchased from the Transfusion Center of the Ulm University Medical Center (IRB granted to the Institut für Klinische Transfusionsmedizin und Immungenetik Ulm GmbH, Ulm, Germany) and were obtained from anonymized healthy blood donors. All blood donors gave written informed consent to authorize the use of their blood for medical, pharmaceutical, and research purposes.

### Cell Cultures

Peripheral blood mononuclear cells (PBMC) from HCMV-seropositive or HCMV-seronegative blood donors (tested by Vidas CMV IgG, BioMérieux, France) were isolated by Ficoll^TM^ (LSM 1077, PAA Laboratories GmbH, Austria) density gradient centrifugation. One portion of the PBMC was used for the immunomagnetic selection of monocytes (Monocyte Isolation Kit II, Miltenyi Biotec, Germany) and the other portion was stored at −80°C in RPMI 1640 (Gibco Life Technologies, Germany) containing 40% fetal bovine serum (FBS) (Sigma-Aldrich Chemie GmbH, Germany) and 10% DMSO (Serva Electrophoresis GmbH, Germany) until needed for stimulation assays of T cells. When specified, T cells were purified from thawed PBMC by negative immunomagnetic selection (Pan T Cell Isolation Kit, Miltenyi Biotec, Germany) following manufacturer’s instructions. M1- or M2-Mφ were generated as previously described (25). Briefly, 3 × 10^6^/ml monocytes were seeded in culture medium [RPMI 1640 supplemented with 10% FBS, 2 mM l-glutamine (Biochrom AG, Berlin, Germany), 100 U/ml penicillin, and 100 U/ml streptomycin (Gibco/BRL)] in hydrophobic Lumox^®^ dishes and differentiated into M1- or M2-Mφ by incubation for 7 days in the presence of 100 ng/ml recombinant human granulocyte-macrophage colony-stimulating factor (GM-CSF) or macrophage-colony stimulating factor (M-CSF) (R&D Systems, Minneapolis, MN), respectively. Human foreskin fibroblasts (HFF) were cultured in minimal essential medium (Gibco Life Technologies) supplemented with 5% FBS, 2 mM l-glutamine, 100 U/ml of penicillin, and 100 U/ml streptomycin. Human umbilical cord endothelial cells (HUVEC) were purchased from Clonetics (BioWhittaker, Walkersville, MA, USA) and cultured in PeproGrow Endothelial Cell basal medium supplemented with 5% fetal calf serum and growth factors (PeproGrow GS-MacroV, Peprotech, Hamburg, Germany).

### Preparation of Viral Stocks and Infection of Mφ Cultures

Cell-free stocks of the endotheliotropic HCMV strain TB40E (32), the recombinant viruses RVTB40/E4ΔUS11 (for brevity later on called ΔUS2-11) (21) and TB40/E-delUL16-green fluorescent protein (for brevity later on called TB40E-GFP) (33, 34) were produced by co-cultivating HFF with late-stage infected HUVEC (HFF:HUVEC ratio of roughly 50:1). Supernatants were harvested from infected cultures showing a 100% cytopathic effect and centrifuged for 10 min at 2,800 × *g* to remove cell debris. Infectious virus was then pelleted by ultracentrifugation for 60 min at 70,000 × *g*, resuspended in 1–2 ml of MEM:sucrose buffer to prevent ice crystal formation, aliquoted, and stored at −80°C. Virus stocks were negative for contamination with *Mycoplasma*, as determined by MicoAlert (Cambrex, Rockland, MD, USA). For determination of the infectious titers, HFF were inoculated with serial dilutions of the virus stocks. After 48 h, the cells were fixed with methanol/acetone (1:1) for 10 min at −20°C and dried completely. Subsequently to a blocking step in PBS 1% milk for 15 min, cells were incubated with antibodies directed against the viral immediate-early proteins 1 and 2 [IE1-2 (35)] for 45 min at 37°C. After washing with PBS, cells were stained with secondary polyclonal rabbit anti-mouse HRP-conjugated antibodies (DAKO, Denmark) and visualized using 3-amino-9-ethylcarbazole (AEC, Sigma-Aldrich Chemie GmbH, Germany) as substrate. IE1-2 positive nuclei were counted and viral infectivity was determined as infectious units (iu) per ml. If not indicated differently, Mφ were infected with a multiplicity of infection (MOI) of 5 iu per cell. When replication-incompetent virus (UV-HCMV) was necessary, the virus DNA was cross-linked by using a CL-1000 Ultraviolet Crosslinker (UVP, UK) with exposure at 200 kJ for 4 min. The efficacy of UV-inactivation was proven at 24 hpi by the lack of IE1-2 positive nuclei in exposed HFF and/or Mφ.

### DNA and RNA Isolation and RT-PCR Analysis

DNA was extracted from TB40E as well as ΔUS2-11 viral particles by using the High Pure Viral Nucleic Acid Kit (Roche, Mannheim, Germany) following the manufacturer’s instructions. 15 ng viral nucleic acids were amplified by PCR using the following primers: IE1-2 (5′-CGTCCTTGACACGATGGAGTC-3′ and 5′-TCGGGGTTCTCGTTGCAATCC-3′) (36), US2 (5′-CAGTCCACAGTCACATACAC-3′ and 5′-GTGATGCCGATCTTCGAGA-3′) (37), US3 (5′-GATGTCGGGCAACTTCAC-3′ and 5′-ACACGCGGCATATTTCTT-3′) (37), US6 (5′-TCGATTCGTATGTTATGCTGC-3′ and 5′-ATCCCGTCCGAACGATAGG-3′) (37), and US11 (5′-TAATAAGTTTGGCATTGGTGG-3′ and 5′-GTCTCCGAAAGCCTCGTC-3′) (37). PCR was repeated for 34 cycles of 1 min at 94°C, 1 min at 57°C, and 1 min at 72°C with a thermal cycler GeneAmp PCR System 9700. For RT-PCR analysis of US2-11 gene expression, 1 × 10^6^ M1- or M2-Mφ were seeded in 24 well plates and infected with an MOI 5 of either TB40E or ΔUS2–11 for 3 h. The unabsorbed virus was inactivated by washing Mφ with citrate buffer (40 nM Na citrate, 10 mM KCl, 135 mM NaCl, pH 3.0) (38) and cells were then maintained in standard medium for the indicated times after infection. Total RNA was extracted from Mφ by using the Qiagen RNeasy Mini kit (Qiagen, Hilden, Germany) following the manufacturer’s instructions. Genomic DNA contaminations were removed by DNase treatment (DNAse I RNAse free, Fermentas now Life Technologies GmbH, Darmstadt, Germany) and cDNA synthesis was performed by using the RevertAid H Minus First strand cDNA synthesis kit (Fermentas now Life Technologies GmbH). RT reaction was performed at 42°C for 60 min. Amplification of the US2, US3, US6, US11, and IE1-2 was performed by using the primers listed above. Amplification of the housekeeping gene glyceraldehyde 3-phosphate dehydrogenase was performed using the following primers forward 5′-TGATGACATCAAGAAGGTGTTGAA-3′ and reverse 5′-TCCTTGGAGGCCATGTGGGCCAT-3′ (Biomol Research Laboratories, Plymouth Meeting, PA, USA). PCR and RT-PCR products were visualized by agarose gel electrophoresis and ethidium bromide staining.

### Immunofluorescence Analyses

Monoclonal antibodies (mAb) reactive against the immediate-early proteins IE72 and IE86 either unlabeled or Alexa488-labeled (mAb E13; Argene-Biosoft, Varilhes, France or MAB810X, clone 8B1.2; Merk Millipore, Darmstadt, Germany, respectively) were chosen to identify infected IE^+^ Mφ. Mφ were seeded in μ-Slide 8 wells (Ibidi, Martinsried, Germany), left uninfected (mock) or infected with either the TB40E or ΔUS2–11 (MOI = 5 if not specified differently), fixed with 4% formaldehyde, permeabilized with 0.2% Triton X-100 and probed with the mAb described above, followed by incubation with Alexa488- or Alexa555-conjugated goat anti-mouse Ig (ICN Biomedical, Eschwege, Germany). Cell nuclei were counterstained with 4′,6′-diamidino-2-phenylindole (DAPI). Staining was detected using a Zeiss Axioskop2 fluorescence microscope (Zeiss, Oberkochen, Germany).

### Flow Cytometry

All flow cytometry stainings were performed according to conventional methods. Cells were harvested, centrifuged, and the pellet was resuspended in PBS containing 3% FBS, 0.01% NaN_3_, and 10% human Ig (Gamunex, Grifols, Germany) to block nonspecific binding sites. The surface staining was performed at 4°C with either primary antibodies or matching isotype controls, as listed below. After 30–60 min the cells were washed in PBS containing 3% FBS and 0.01% NaN_3_, centrifuged, either resuspended in the same buffer or fixed with 1% paraformaldehyde and kept at 4°C until acquisition. The HCMV IE1-2 and MHC class I and II double stainings were performed by labeling MHC on the surface of viable Mφ, followed by fixation (4% paraformaldehyde), permeabilization (triton 0.2%), and incubation with either the directly labeled anti CMV antibody described above or the Alexa488 corresponding isotype control. For the T-cell activation assays, fixation, permeabilization, and intracellular cytokine staining were performed using BD Cytofix/Cytoperm^TM^ Kit (BD Biosciences) according to the manufacturer’s manual. The following monoclonal mouse antibodies and matching isotype controls were used: FITC-anti-CD4 (RPA-T4), FITC-anti-CD107a (H4A3), FITC-IgG1 (MOPC-21), FITC- or PE-anti-HLA-A,B,C (G46-2.6), FITC- or PE-anti-HLA-DR (TU36), FITC/PE-IgG1, PE-IgG1 (MOPC-21), PE-IgG2b, PerCP-anti-CD69 (L78) and PerCP-IgG1 (MOPC-21), and finally Allophycocyanin-anti-CD56 (B159) all from BD Biosciences. Allophycocyanin-anti-CD8 (BW135/80), allophycocyanin-IgG2a (S43.10), APC-IgG2a, PE-anti-HLA-DR (AC122), PE-anti-CCR7 (REA108), PE-anti-CD4 (M2-T466), PE-anti-CD19 (LT19), PE-anti-IFN-γ (45-15), PE-IgG1 (IS11-12E4.23.20), PE-IgG1, PE-IgG2a, PerCP-anti-CD45RA (T6D11), and PerCP-IgG2b (IS6-11E5.11) all from Miltenyi Biotec. Allophycocyanin-anti-CD4 (EDU-2) and allophycocyanin-IgG2a (PPV-04) purchased from ImmunoTools (Germany).

### Cell Sorting of HCMV-Infected Mφ

M2-Mφ were seeded into Lumox^®^ 24 well plates (Sarstedt AG & Co., Germany) and either mock or TB40E-GFP infected (MOI = 5) for 24 h. Cells were then harvested, resuspended in PBS at the concentration of 6 × 10^6^/ml and sorted using a FACSAria III flow cytometer (BD Biosciences). Cells were initially gated on the basis of forward and side scatter (live), the live cells were then first gated to eliminate doublets, and finally by positive GFP signal. The instrument setting was performed using M2-Mφ inoculated with the UV-treated and replication incompetent TB40E-GFP as negative control.

### Western Blot

2.5 × 10^5^ cells (either unsorted, GFP^+^ sorted and GFP^–^ sorted Mφ) were lysed in 100 µl ROTI-1 sample buffer, sonicated and boiled at 95°C for 5 min prior to electrophoresis using 7.5% SDS-polyacrylamide gels. After separation, proteins were transferred to nitrocellulose membranes (Biorad) by the semidry method. Free binding sites on the nitrocellulose membranes were blocked by incubation in 5% skim milk in PBS containing 0.3% Tween 20 before the antibodies (diluted in PBS-0.3% Tween 20 with 0.5% skim milk) were added. Bound antibodies were detected with goat anti-mouse horseradish peroxidase-conjugated antibodies (pierce) and visualized by enhanced chemiluminescence.

### T-Cell Proliferation Assay

M1- and M2-Mφ were seeded into Lumox^®^ 24 well plates (Sarstedt AG & Co.) and inoculated with an MOI of 5 of either TB40E or ΔUS2-11, both of them used either as infectious or replication-incompetent upon UV inactivation. Additionally, M1- and M2-Mφ were treated with 50 µg/ml tetanus toxoid (TT; Statens Serum Institute, Denmark) or left untreated. After 1 day, cells were harvested and resuspended in RPMI containing 5% of human AB serum (HS) obtained from HCMV-seronegative donors (HS; Institut für Klinische Transfusionsmedizin und Immungenetik Ulm GmbH) and sublethally irradiated (3000 cGy) in order to prevent cellular replication. Autologous PBMC were thawed, labeled with CFSE Cell Division Tracker Kit (Biolegend, Fell, Germany) according to the manufacturer’s instructions and resuspended in RPMI 5% HS. Mφ were distributed into 96 well microplates with U-bottom (Greiner Bio-One GmbH, Germany) prior to addition of CFSE-labeled PBMC or CFSE-labeled purified T cells at a Mφ/PBMC ratio of 1:8 and Mφ/T cell ratio of 1:4. As controls, PBMC alone were left untreated or stimulated with 5 µg/ml phytohemagglutinin-L (eBioscience, Frankfurt am Main, Germany). After 6 days, PBMC or T cells were harvested and stained with antibodies directed against CD4, CD8, CD19, CD56, CCR7, and CD45RA as described above. After gating for lymphocyte-morphology, the percentage of proliferating cells was calculated gating for CFSE^low^ cells within the total CD4^+^ or CD8^+^ T-cell populations.

### T-Cell Activation and Degranulation Assays

M1- and M2-Mφ were seeded into 96 well microplates (Greiner Bio-One GmbH) and were infected with TB40E, ΔUS2-11 (MOI of 5) or left untreated. After 24 h, Mφ were co-cultured with autologous PBMC at a ratio 1:10 for further 18 h. For T-cell activation assays, 1 µg/ml brefeldin A (Sigma-Aldrich Chemie GmbH) was added after 18 h of Mφ/PBMC co-culture to all conditions for 4 h to prevent cytokine release. PBMC were then collected, permeabilized, stained with antibodies directed against CD4, CD8, CD69, and IFN-γ, and analyzed by flow cytometry as described above. For T-cell degranulation assays, 10 µl/ml monensin (BD GolgiStop™, BD Biosciences) was added after 48 h of Mφ/PBMC co-culture together with either antibodies directed against CD107a or corresponding isotype control. After 4 h of incubation at 37°C, PBMC were collected, stained with antibodies directed against CD8, and analyzed by flow cytometry as described above.

### Statistical Analysis

Statistical analysis was performed using GraphPad Prism 5 Software (GraphPad Software, Inc., USA). To define statistically significant differences comparing a single variable between paired samples, either a two-tailed paired Student’s *t*-test or a Wilcoxon matched-pairs test was performed, depending if Gaussian distribution could be assumed or not. Kruskal–Wallis and Dunn’s multiple comparison tests were performed if Gaussian distribution was not applicable and if a single variable should be compared among more than two unmatched groups. *p* Values were considered significant if <0.05.

## Results

### US2, US3, US6, and US11 Genes Are Expressed in TB40E-Infected M1- and M2-Mφ with an Immediate-Early Kinetic

**Figure 1:**
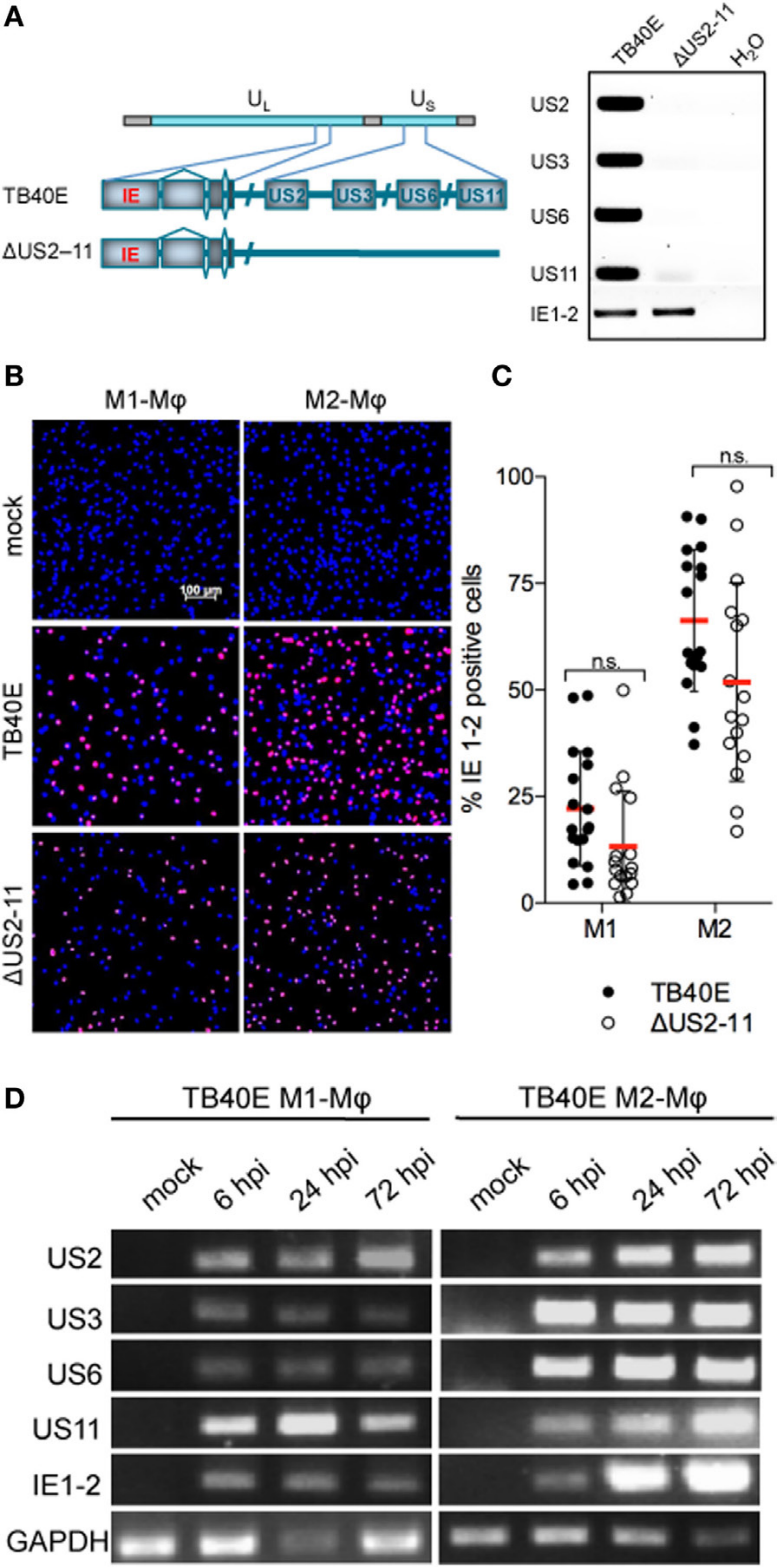
Mφ infected by wild-type TB40E, but not ΔUS2-11 mutant express the immune evasive genes US2, US3, US6, and US11 with an immediate-early kinetic. **(A)** Schematic representation of the US2, US3, US6, and US11 gene deletion in the context of the whole TB40E genome. On the right, PCR amplification of US2, US3, US6, US11, and IE1-2 genes using as template viral DNA extracted from viral particles of the wild type TB40E or its derivative RVTB40/E4ΔUS11 virus (ΔUS2–11). **(B,C)** Expression of IE1-2 proteins in M1- and M2-Mφ infected with TB40E or ΔUS2–11 (MOI = 5). **(B)** At 24 hpi, cells were fixed, stained with primary antibodies detecting the viral IE1-2 proteins and secondary AlexaFluor555-conjugated antibodies. Nuclei were counterstained with DAPI. Pictures were taken at 10x magnification and the percentage of IE1-2 positive cells was calculated. **(C)** Each symbol represents cells obtained from one blood donor. Bars represent mean values ± standard error of the mean (SEM). *p* calculated with Student’s two-sample equal variance *t*-test, with a two-tailed distribution; n.s., not significant. **(D)** M1- and M2-Mφ were infected with TB40E (MOI = 5) for 3 h or left untreated (mock). Total RNA was isolated at the indicated time points and analyzed by RT-PCR. Gene products corresponding to the indicated viral genes (US2 231 bp; US3 227 bp; US6 260 bp; US11 113 bp; and IE1-2 300 bp) as well as the cellular houskeeping gene glyceraldehyde 3-phosphate dehydrogenase (GAPDH, 250 bp) were visualized by agarose gel electrophoresis. Results are from one donor representative of three.

M1- and M2-Mφ were infected with an MOI 5 of either the wild-type HCMV TB40E or the recombinant virus RVTB40/E4ΔUS11, lacking the immune evasive genes US2, US3, US6, and US11 (21), for brevity later on called ΔUS2-11(Figure 1A). At different time points after infection, Mφ infection rates were calculated by quantifying the percentage of cells expressing the viral immediate-early proteins 1-2 (IE1-2) (Figure 1B). The deletion of the four immune evasive genes did not modify virus infectivity and wild-type TB40E and ΔUS2-11 viruses infected comparable amounts of Mφ at 1 as well as 3 days post infection (dpi) (Figure 1C and data not shown). M2-Mφ were more susceptible to HCMV infection than M1-Mφ, irrespectively, on the presence or lack of the four immune evasive genes. Starting at 6 hpi and persistently until 72 hpi, transcripts specific for the immune evasive genes US2, US3, US6, and US11 were detected by RT-PCR in TB40E-infected Mφ but not in mock-infected cells (Figure 1D) or in Mφ infected with ΔUS2-11 (data not shown). The PCR products did not originate from contaminating viral DNA but truly reflected mRNA transcripts as proven by the lack of PCR products in the absence of retrotranscriptase enzyme as well as by the lack of PCR products in Mφ treated with the replication incompetent UV-inactivated TB40E (UV-TB40E) (data not shown).

### US2, US3, US6, and US11 Genes Counteract MHC Class I Upregulation That Follows TB40E Infection of Mφ

**Figure 2:**
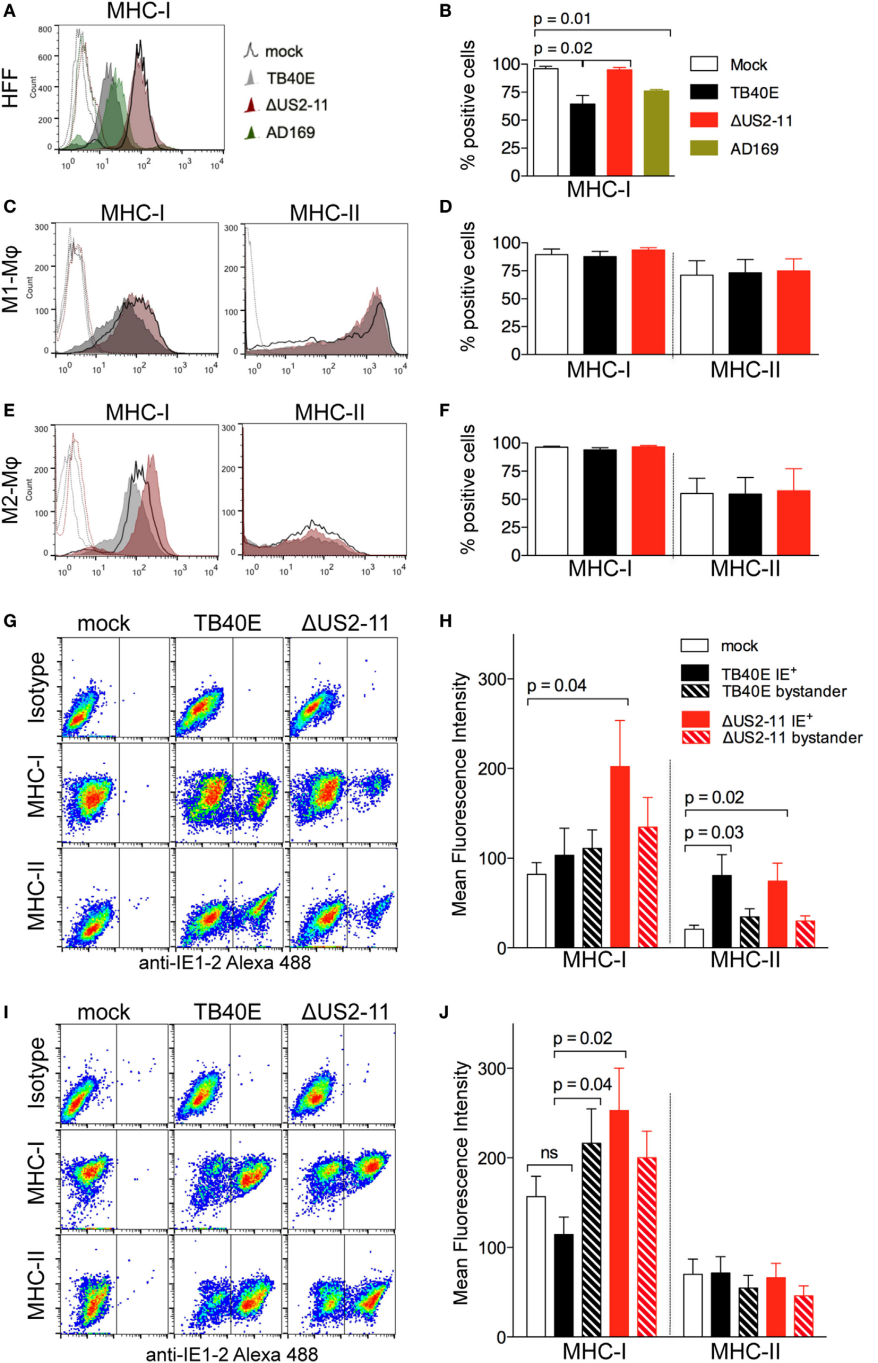
At 1 dpi, infected and bystander Mφ do not undergo major histocompatibility complex (MHC) class I and class II downregulation and maintain MHC class I and class II expression levels comparable or even higher than mock-infected cells. Human foreskin fibroblasts (HFF), M1- and M2-Mφ were left untreated (mock) or infected with an MOI 5 of the wild-type TB40E, the mutant ΔUS2–11 or the fibroblast-adapted HCMV strain AD169. At 1 dpi, viable HFF **(A,B)**, M1-Mφ **(C,D)** and M2-Mφ **(E,F)** were labeled with anti-MHC-I, anti-MHC-II or isotypic control antibodies and examined by flow cytometry. Bars represent mean values ± SEM obtained from three HFF **(B)** and five M1- and M2-Mφ experiments **(D,F)**. *p* calculated with Student’s two-sample equal variance *t*-test, with a two-tailed distribution. At 1 dpi, M1-Mφ **(G,H)** and M2-Mφ **(I,J)** were firstly incubated with PE-labeled anti-MHC-I, anti-MHC-II or isotypic control antibodies, then fixed, permeabilized, incubated with the Alexa488-labeled anti-IE1-2 antibodies, and examined by flow cytometry. Bars represent mean fluorescence intensity values ± SEM obtained with cells from five different blood donors **(H,J)**. *p* Calculated with Student’s two-sample equal variance *t*-test, with a two-tailed distribution.

At first, we tested whether the ΔUS2–11 virus had effectively lost the capacity to downregulate MHC class I molecules in infected cells. At 1 dpi, while HFF infected with TB40E or AD169 HCMV strains (both possessing the four immune evasive genes US2-11) expressed lower levels of MHC class I molecules than mock-infected cells (Figures 2A,B), no MHC class I molecule downregulation was observed in HFF infected by ΔUS2-11 virus. In contrast to HFF, in both M1- and M2-Mφ (Figures 2C–F) the expression levels of MHC class I as well as class II molecules remained comparable in TB40E-, ΔUS2–11- and mock-infected cells thus suggesting that US2-11 immune evasive genes may have only a limited effect in these cells.

In order to address whether MHC downregulation in infected Mφ could be masked by the concomitant upregulation of MHC molecules on neighboring bystander cells, a phenomenon already reported for HCMV-infected HFF (39, 40), we performed flow cytometry double staining of the viral IE1-2 proteins and MHC class I or class II molecules. As shown in Figures 2G,H for M1-Mφ, upon TB40E infection MHC class I molecules were expressed at comparable levels in IE^+^, mock-infected as well as bystander cells. In the absence of US2-11 genes, IE^+^ cells expressed higher levels of MHC class I molecules, thus suggesting that upon HCMV infection Mφ undergo MHC class I upregulation but this effect is efficiently counteracted by the US2-11 genes. Similarly, TB40E IE^+^ M2-Mφ (Figures 2I,J) maintained MHC class I levels that were comparable to mock-infected cells, while ΔUS2–11 IE^+^ Mφ expressed higher MHC class I levels, thus confirming that also in M2-Mφ the four immune evasive genes counterbalance MHC upregulation induced by infection. In contrast to MHC class I, MHC class II molecules were unaffected by US2-11 gene products and remained similarly expressed in both TB40E and ΔUS2–11 IE^+^ Mφ at levels that were similar or higher than those of mock-infected cells (Figures 2H,J).

To address whether the US2-11 mediated inhibition of MHC molecules might be more prominent at later stages of infection, we performed a similar analysis at 3 dpi. While in infected HFF there was an almost complete downregulation of MHC class I molecules at 3 dpi, infected Mφ cultures maintained considerable levels of MHC class I and class II molecule expression on cell surface (Figure S1A in Supplementary Material). The viral inhibitory effect on MHC expression was present when the cells were infected with the wild-type TB40E, but not with the virus lacking the US2-11 genes, and infection with ΔUS2-11 virus led to MHC-I levels comparable to (in HFF and M2-Mφ) or even higher (in M1-Mφ) than those of uninfected cells. The data collected at 3 dpi resemble those obtained at 1 dpi and confirm that the four immune evasive genes counterbalance the MHC upregulation induced by HCMV infection in Mφ. This interpretation is supported by the differential analysis of IE^+^ and IE^–^ bystander cells within the infected Mφ cultures (Figures S1B,C in Supplementary Material). IE^+^ Mφ infected with the ΔUS2–11 virus exhibited higher levels of MHC class I molecules than uninfected cells and IE^+^ cells infected with the wild-type TB40E thus suggesting that Mφ respond to HCMV infection with MHC-I upregulation, but this effect is efficiently counteracted by the pUS2-11. However, the efficiency of the US2-11 gene products appears more prominent at later time points in HCMV-infected Mφ cultures.

All together our data demonstrate that upon HCMV infection Mφ do not undergo the dramatic inhibition of MHC class I and II molecules already reported in HCMV-infected HFF or DC (21, 41), but remain capable to present MHC class I and class II molecules on the cell surface at 1 dpi as well as 3 dpi.

### Effector and Central Memory CD4^+^ and CD8^+^ T Cells Vigorously Proliferate in Response to TB40E or ΔUS2–11-Infected Mφ

**Figure 3:**
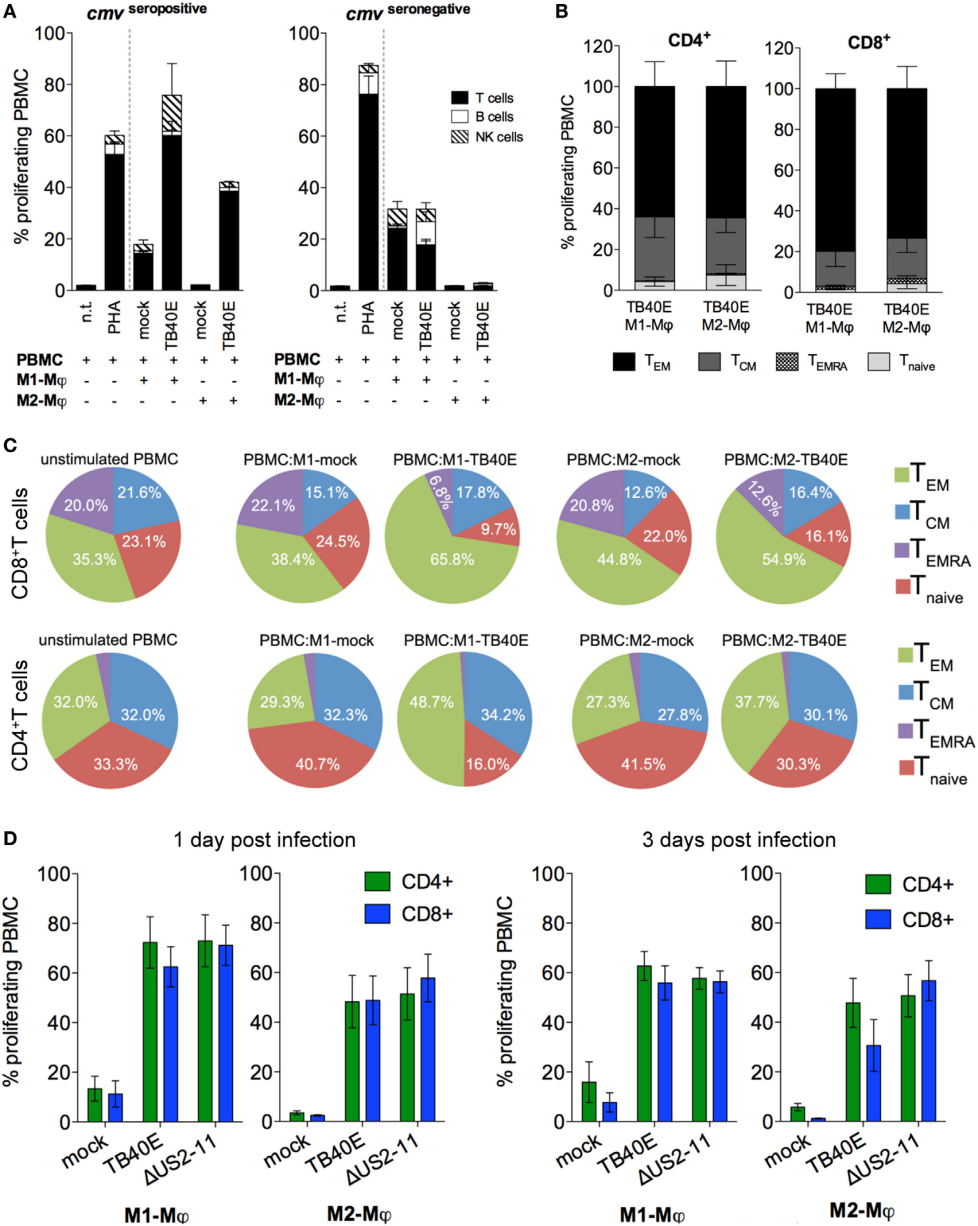
The proliferation of memory CD4^+^ and CD8^+^ T cells elicited by TB40E-infected M1- and M2-Mφ is not inhibited by the immune evasive genes US2-11. M1- and M2-Mφ derived from HCMV-seronegative **(A)** or HCMV-seropositive **(A–D)** blood donors were left untreated (mock) or infected with an MOI of 5 of the wild-type TB40E or the mutant ΔUS2–11. **(A,B)** After 1 day, M1- and M2-Mφ were harvested, irradiated (30 cGy) and added as stimulators in a ratio of 1:8 to autologous CFSE-labeled PBMC for 6 days. As controls, CFSE-labeled PBMC alone were left untreated (n.t.) or stimulated with 5 µg/ml phytohemagglutinin (PHA). **(A)** PBMC were harvested, stained with fluorescently labeled antibodies directed against CD4, CD8, CD19, and CD56 and subsequently analyzed by flow cytometry. Bars represent mean values ± SEM from 5 HCMV-seronegative and 7 HCMV-seropositive blood donors. **(B)** PBMC were collected and stained with fluorescently labeled antibodies directed against CD4, CD8, CCR7, CD45RA, and subsequently analyzed by flow cytometry. The proliferating cells were classified into effector memory (T_EM_; CD45RA^−^ CCR7^−^), central memory (T_CM_; CD45RA^−^ CCR7^+^), revertant effector memory (T_EMRA_; CD45RA^+^ CCR7^−^) and naïve (T_naive_; CD45RA^+^ CCR7^+^) T cells. Bars represent mean values ± SD obtained from cells of four donors. **(C)** Pie charts depict the phenotypic changes induced by HCMV-infected Mφ in the composition of the CD8^+^ and CD4^+^ populations. The main T cell subsets are indicated by the adjoining key. Mean values were obtained from four independent experiments. **(D)** At 1 and 3 dpi, Mφ were harvested and co-cultivated with autologous PBMC as described above. Bars represent mean values ± SEM obtained from 5 blood donors.

An important consequence of the presence of MHC class I and II molecules on the surface of an infected cell is the presentation of viral antigens to antigen-specific T cells. As shown in Figure 3A, when the cells were obtained from HCMV-seropositive but not from HCMV-seronegative blood donors, TB40E-infected M1- and M2-Mφ induced high proliferative responses in autologous T cells. Lower proliferative responses were also elicited in NK cells, but not in B cells. By focusing on cells obtained from HCMV-seropositive donors and by using CCR7 and CD45RA as discriminatory markers between naïve and memory T cells (42), we could characterize the proliferating CD4^+^ and CD8^+^ T cells as mainly being effector memory (T_EM_; CD45RA^−^ CCR7^−^) or central memory (T_CM_; CD45RA^−^ CCR7^+^) T cells (Figure 3B). Only very small proportions of naïve T cells (T_naive_; CD45RA^+^ CCR7^+^) and effector memory RA T cells (T_EMRA_; CD45RA^+^ CCR7^−^) (43–45) were detected among the proliferating cells in response to Mφ stimulation in our experimental setting. During the 6 days co-culture, TB40E-infected Mφ shaped the T cell composition within the PBMC. As shown in Figure 3C, in both CD4^+^ and CD8^+^ compartments the T_EM_ and T_CM_ subpopulations enlarged at the expenses of T_naive_ and T_EMRA_. At the end of the culture, T_EM_ and T_CM_ reached an average of 60 and 17% of the total CD8^+^ T cells, and an average of 43 and 32% of the total CD4^+^ T cells. In contrast, when mock-infected M1- or M2-Mφ were used as stimulators, the T-cell composition within the PBMC remained unchanged as compared to the beginning of the co-culture.

The immune evasive genes US2, US3, US6, and US11 had little or no functional relevance on T-cell-stimulatory potential of M1- and M2-Mφ. As shown in Figure 3D, comparable CD4^+^ and CD8^+^ T-cell proliferative responses were triggered by TB40E-infected or ΔUS2–11-infected Mφ at all tested time points after infection.

### Both IE^+^ and Bystander Mφ Present Viral Antigens to CD4^+^ and CD8^+^ T Cells

**Figure 4:**
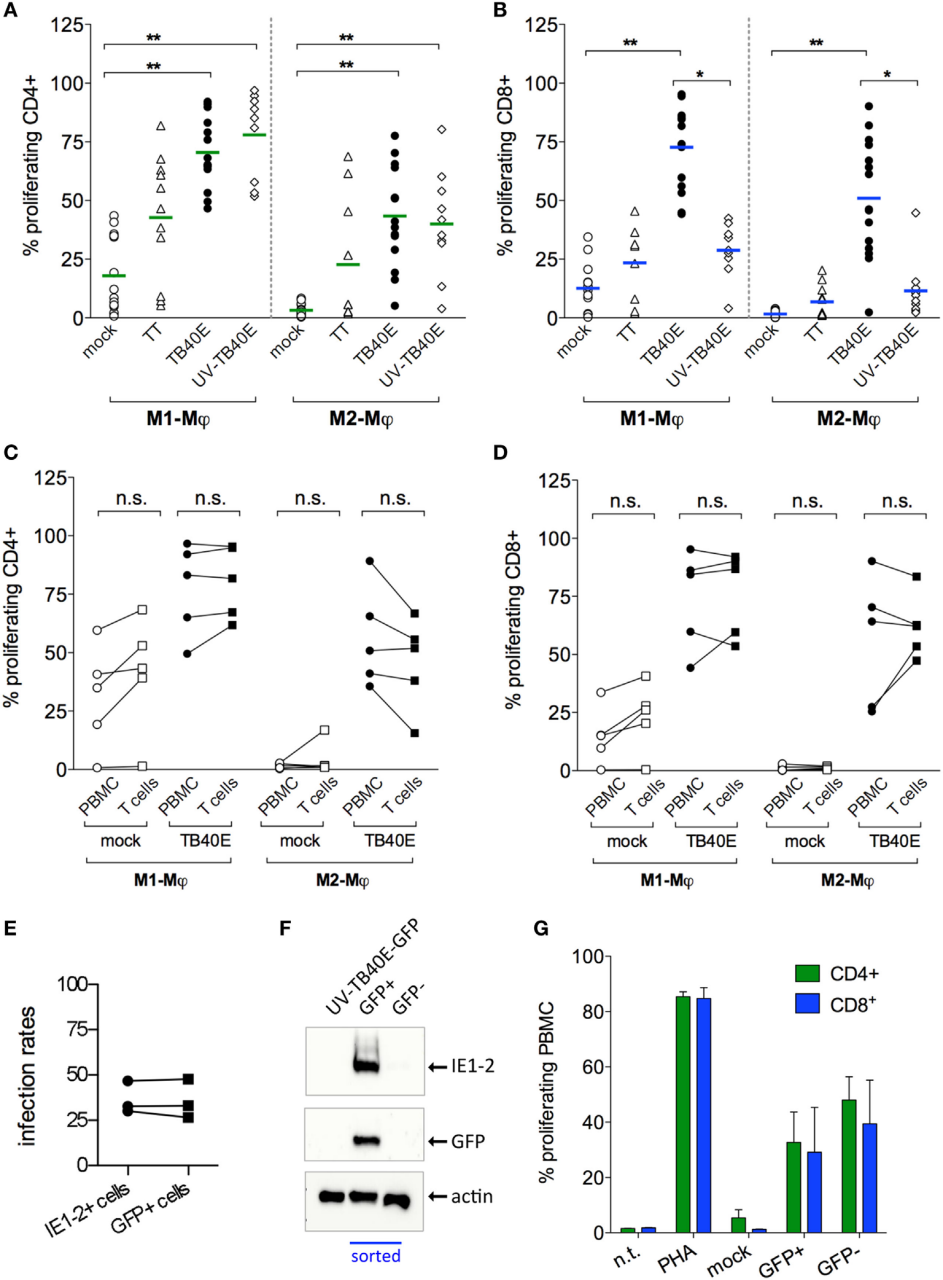
Infected and bystander Mφ cooperate for direct and cross presentation of viral antigens to T cells. M1- and M2-Mφ derived from HCMV-seropositive blood donors were inoculated with an MOI of 5 of either replication-competent TB40E (TB40E) or replication incompetent UV-inactivated TB40E (UV-TB40E), treated with 50 µg/ml tetanus toxoid (TT), or left untreated (mock). **(A,B)** At 1 dpi, Mφ were harvested, irradiated, and co-cultivated with autologous CFSE-labeled PBMC for 6 days. Percentages of proliferating (CFSE^low^) cells within the CD4^+^ and CD8^+^ populations are depicted. Each dot represents cells obtained from one blood donor and mean values are depicted as lines. *p < 0.05, **p < 0.01 (Kruskal–Wallis test and Dunn’s multiple comparison test). **(C,D)** At 1 dpi, mock-infected and TB40E-infected Mφ were harvested, irradiated, and added as stimulators to either autologous CFSE-labeled purified T cells (T cells; ratio of 1:4) or to unfractionated CFSE-labeled PBMC (PBMC; ratio of 1:8) for 6 days. Percentages of proliferating (CFSE^low^) cells within the CD4^+^ and CD8^+^ populations are depicted. Cells derived from the same donor are connected by a line. n.s., not significant (Wilcoxon matched-pairs test). **(E,F)** M2-Mφ were inoculated with an MOI of 5 of the green fluorescent virus TB40E-GFP (either replication-competent or UV-inactivated) or left untreated (mock) for 24 h. Cells were then harvested and sorted by positive GFP signal using a FACSaria flow cytometer. **(E)** Comparison of the infection rates measured by GFP^+^ expression at the cell sorter (GFP^+^ cells) and by indirect immunofluorescence analysis after viral IE1-2 proteins staining (IE1-2+ cells) as described in Figure 1. **(F)** GFP^+^ and GFP^−^ sorted Mφ as well as not-sorted Mφ treated with UV-TB40E-GFP were lysed in Roti Buffer (2.5 × 10^5^ cells/100 μl) and analyzed by SDS-PAGE using monoclonal antibodies against the viral IE1-2 proteins, actin and GFP. Western blot of one donor representative of three is shown. **(G)** Mock-infected, GFP^+^ and GFP^–^ sorted Mφ were co-cultivated with autologous CFSE-labeled PBMC. After 6 days, the percentage of CFSE^low^ proliferating CD4^+^ and CD8^+^ T cells was quantified by flow cytometry. Bars represent mean values ± SEM from 3 blood donors.

Since the antigen presentation pathways used by HCMV-infected Mφ are still largely unknown, we decided to investigate the extent of Mφ direct and cross-presentation of viral antigens. As shown in Figure 4A, M1- and M2-Mφ loaded with UV-TB40E as well as actively infected by TB40E induced comparable CD4^+^ T-cell proliferative responses, thus indicating that MHC class II dependent presentation of exogenous viral antigen to CD4^+^ T cells is constitutively high in Mφ and is not enhanced by active infection. On the other hand (Figure 4B), while MHC class I dependent presentation to CD8^+^ T cells was optimal when M1- and M2-Mφ were actively infected, only low levels of CD8^+^ T-cell proliferation were induced by Mφ loaded with the UV-TB40E or with the soluble TT. To exclude that APC other than Mφ could account for the observed T-cell stimulation, responder T cells were purified from the PBMC and co-cultivated with autologous mock-infected or TB40E-infected Mφ as previously described. As shown in Figures 4C,D for CD4^+^ and CD8^+^ T cells, respectively, comparable proliferating cells were detected in responder unfractionated PBMC or responder purified T cells, thus demonstrating that Mφ and no other APC were necessary and sufficient for the induction of T-cell responses.

All together these findings suggest that while helper cells respond comparably to actively infected and antigen-loaded Mφ, high cytotoxic proliferative responses require the active infection of Mφ.

In order to investigate the reciprocal contribution of IE^+^ and bystander Mφ to the induction of T-cell proliferation, IE^+^-infected Mφ were sorted from the IE^–^ bystander Mφ prior to co-culture with autologous PBMC and measurement of T-cell proliferation (Figures 4E–G). Due to their higher susceptibility to HCMV infection and to the possibility to collect higher numbers of IE^+^ cells, M2-Mφ were chosen instead of M1-Mφ for these assays. The recombinant TB40/E-delUL16-GFP (for brevity later on called TB40E-GFP), expressing GFP under the control of the UL16 gene promoter and possessing an intact US2-11 genetic locus (33), was selected due to its bright GFP expression, its wild type-alike infectivity (34) and for the perfect correspondence between the GFP signal and the expression of IE1-2 proteins (Figures 4E,F). Sorted GFP^+^ infected Mφ elicited comparable CD4^+^ and CD8^+^ T cells than GFP^–^ bystander Mφ (Figure 4G), thus demonstrating that (i) both infected and bystander Mφ concur to viral antigen presentation and induction of T-cell responses and (ii) exogenous viral peptides present in the GFP^-^ bystander Mφ can also be cross-presented to CD8^+^ T cells.

### CD4^+^ and CD8^+^ T Cells Are Functionally Activated by HCMV-Infected Mφ

**Figure 5:**
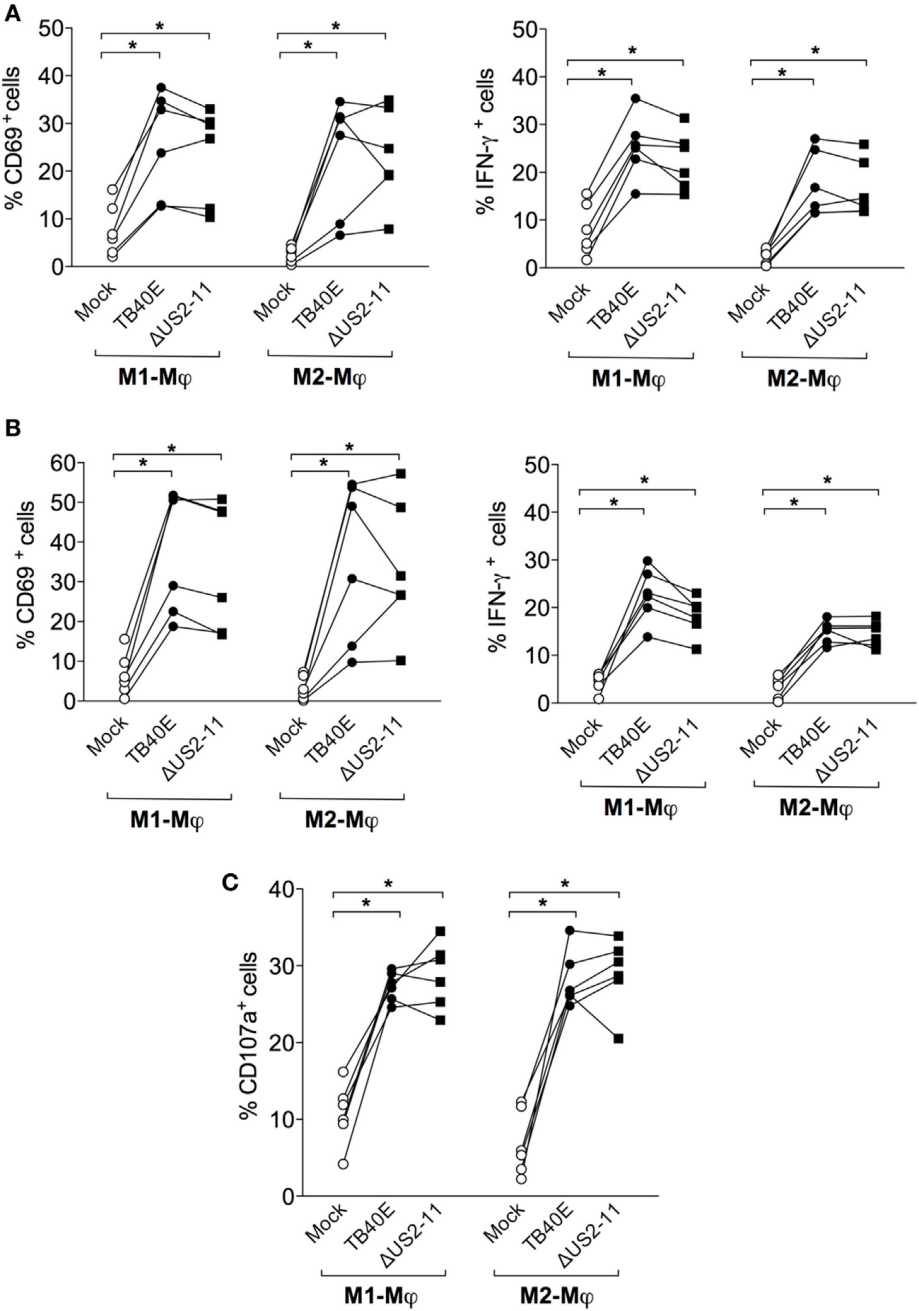
HCMV-infected Mφ induce functional activation of CD4^+^ and CD8^+^ T cells. M1- and M2-Mφ derived from HCMV-seropositive blood donors were infected with an MOI of 5 of the wild-type TB40E, the mutant ΔUS2–11 or left untreated (Mock). **(A,B)** After 1 day, Mφ were co-cultured with autologous PBMC for 18 h prior to addition of 1 µg/ml Brefeldin A. Four hours later, PBMC were harvested, permeabilized, and stained with antibodies directed against CD4, CD8, CD69, and IFN-γ. Percentages of CD4^+^ **(A)** and CD8^+^ **(B)** T cells expressing CD69^+^ and IFN-γ^+^ are depicted. Cells derived from the same donor are indicated by a line. **(C)** After 1 day, Mφ were co-cultured with autologous PBMC for 48 hours. Cells were then incubated with monensin and a fluorescently labeled antibody directed against CD107a for 4 h. Subsequently, PBMC were stained with antibodies directed against CD8. Percentages of CD107a^+^ cells among the CD8^+^ T cells are depicted. Cells derived from the same donor are connected by a line. *p < 0.05, **p < 0.01 (Wilcoxon matched-pairs test).

Protective immunity against HCMV requires not only a sufficient number of HCMV-specific T cells but also T cells capable of executing multiple effector functions. First, we assessed whether upon encounter with wild type TB40E or mutant ΔUS2–11-infected Mφ T cells expressed the activation marker CD69 (46) and/or produced IFN-γ (47, 48). As shown in Figures 5A,B, CD4^+^ and CD8^+^ T cells became activated and produced IFN-γ upon co-culture with TB40E-infected and ΔUS2–11 infected, but not with mock-infected Mφ. Even though TB40E-infected M1-Mφ exhibited slightly higher stimulation capacities than TB40E-infected M2-Mφ, an average of 21% of CD4^+^ T cells and 19% of CD8^+^ T cells produced IFN-γ in response to HCMV-infected autologous Mφ. The immune evasive genes US2, US3, US6, and US11 expressed by infected Mφ had no impact on the T-cell activation. Finally, to assess whether HCMV-infected Mφ could induce cytotoxic CD8^+^ T-cell activation, we measured the T-cell capacity to expose the lytic granule marker CD107a (LAMP-1) on the cell surface (49). As shown in Figure 5C, stimulation with both TB40E and ΔUS2–11, but not with mock-infected Mφ induced the degranulation of 27% CD8^+^ T cells. Interestingly, whereas Mφ challenged with UV-TB40E could stimulate CD4^+^ T cells in the same extent than TB40E-infected Mφ, the expression levels of CD107a on CD8^+^ T cells were less elevated when Mφ were challenged with UV-TB40E as compared with TB40E-infected Mφ (data not shown). All together these data reveal that T cells respond to stimulation by TB40E-infected Mφ by exhibiting features of immune activation and acquiring both cytokine secretory functions as well as increased expression levels of the lytic granule marker CD107a.

## Discussion

In healthy HCMV-seropositive individuals—corresponding to 40–100% of the human population—the memory T-cell pool includes a large amount of HCMV-specific CD4^+^ and CD8^+^ T cells, which can exceed 10% of the total T cells in middle aged adults and even reach 50% in elderly people (11, 50–52). These HCMV-specific T cells are mainly classified as effector memory T cells, either classical effector memory (T_EM_) or terminally differentiated effector T cells re-expressing CD45RA (T_EMRA_) (20) and are essential for controlling and restricting viral replication (53–57). When stimulated *ex vivo* with HCMV peptides, the HCMV-specific T memory cells express the activation marker CD69, release the inflammatory cytokine IFN-γ, and exhibit cytotoxic potential (11, 47, 48). It has been hypothesized that such a large and sustained HCMV-specific T-cell response is maintained through a steady boost of the immune system with viral antigens produced during frequent viral reactivations and low-grade HCMV replication events (13). However, while CD4^+^ T cells can be stimulated by MHC class II-expressing cells that have simply taken up and processed infected cells or viral particles, CD8^+^ T cells require presentation of viral antigen in association with MHC class I molecules on the surface of infected cells.

In this regard, it is somewhat of a paradox that HCMV is generally paradigm for immune evasion. Between the numerous immune interfering genes ascribed to HCMV, the US region of the HCMV genome encodes at least four glycoproteins that inhibit MHC class I endogenous and MHC class II exogenous antigen-presenting/processing pathways [reviewed in Ref. (15, 17)]. Several *in vitro* studies performed with transfected cell lines as well as HCMV-infected HFF have demonstrated that the US2, US3, US6, and US11 gene products are each independently sufficient to downmodulate MHC class I (58–61) and MHC class II molecules (62–64). Moreover, the most potent APC the DC are immunologically paralyzed by HCMV infection (22) [reviewed in Ref. (16, 65)] and it has been extensively reported that upon HCMV infection DC become unable to stimulate CD8^+^ and CD4^+^ T cells (21, 41, 66, 67).

To solve the paradox of a large HCMV-specific T-cell pool and the presence of dysfunctional DC, two alternative mechanisms have been hypothesized. On the one hand, it has been proposed that uninfected DC could take up viral antigens and cross-present them in complex with MHC class I molecules to CD8^+^ T cells (68, 69). On the other hand, it has been suggested that since blood CD11c^+^ DC are not impaired by HCMV, they could account for the anti-HCMV T-cell response (70). Keeping in mind that the HCMV-specific T-cell response is on the same time extraordinary ample and considerably diverse (11, 71, 72), it is likely that these two compensatory mechanisms might be insufficient. On the one hand, blood CD11c^+^ DC are largely immature (73) and account for less than 1% of circulating leukocytes (70) and on the other hand, the real biological relevance of cross-presentation for induction and maintenance of adaptive immune responses is still controversial (68, 74).

We therefore hypothesized that Mφ, the second most potent APC subset (30), could be refractory to HCMV-mediate downregulation of MHC molecules and remain capable of both efficient viral antigen presentation and T-cell stimulation after HCMV infection. Several evidences support this hypothesis. In the murine experimental model of cytomegalovirus infection, it has been shown that upon MCMV infection primary Mφ express and present viral antigens in association with MHC class I molecules and efficiently stimulate T cells (23). Moreover, Mφ support a persistent infection with low-grade release of viral progenies (24–26) and during natural infection Mφ accumulating in areas of infection express viral proteins (27).

Since we previously reported that after HCMV infection M1- and M2-Mφ exhibit features of classical activation, expressing high levels of co-stimulatory and MHC class I molecules and secreting high amounts of inflammatory cytokines and chemokines (25), we decided to investigate (i) whether the immune evasive genes US2, US3, US6, and US11 were expressed during HCMV infection in Mφ; (ii) which impact these genes exerted on the expression and function of MHC class I and class II molecules, and (iii) whether IE^+^ infected Mφ could directly present antigens to T cells.

By using the US2, US3, US6, and US11 primer pairs applied by Park and colleagues to study the temporal pattern of US genes expression in HFF (37), we detected transcripts of the four immune evasive genes starting as early as 6 hpi and persistently until 72 hpi in TB40E-infected Mφ. Differently from HFF, where the expression of US3 was transient (37), in M1- and M2-Mφ all four immune evasive genes were expressed during the entire time of observation. In contrast to HFF and DC (21, 66, 75), in which TB40E, but not the ΔUS2–11 mutant induced a prompt and robust downregulation of MHC molecules, expression levels of MHC class I and class II molecules in Mφ infected with TB40E were comparable to uninfected cells. Since IE^+^ Mφ infected with ΔUS2–11 expressed higher levels of MHC class I molecules than uninfected cells, we believe that HCMV infection leads to the upregulation of MHC class I molecules in Mφ and that the role of the immune evasive US2-11 gene products is to compensate this effect.

Similarly to MCMV-infected murine Mφ (23), TB40-infected human Mφ remain capable to present MHC molecules on the cell surface despite the expression of viral MHC antagonists. Different mechanisms might account for the incomplete inhibitory capacity of pUS2-11 in Mφ. For example, the abundance and kinetic of pUS2-11 may be suboptimal in Mφ as compared to the more permissive HFF. In this scenario, the high amount of antigen-MHC class I complexes formed in TB40E-infected Mφ might exhaust the inhibitory capacity of the US2-11 immune evasive genes and thus results in the preservation of sufficient antigen-MHC class I complexes for recognition by high-avidity CD8^+^ T cells. As an alternative explanation, pUS2-11 function could be hampered in infected Mφ by an insufficient expression of essential pUS2-11 co-factors such as the E2 and E3 ubiquitin ligases UBE2G2 (76), TRC8 (77), and TMEM129 (78, 79).

Professional APC can use a variety of mechanisms to process and present antigenic proteins; typically, while MHC class I molecules bind endogenous peptides derived from proteins expressed inside the cell, MHC class II molecules bind exogenous peptides derived from ingested proteins (80). In addition, professional APC can cross-present exogenous proteins in complex with MHC class I (81, 82) and thus stimulate CD8^+^ T cell without being infected. As mentioned before, this last mechanism is considered essential for the generation of HCMV-specific T cells when HCMV-infected DC are immunologically dysfunctional. Since the antigen presentation pathways used by Mφ are still largely unknown, we decided to evaluate the direct and cross-presentation of HCMV antigens in M1- and M2-Mφ. Since Mφ treated with replication-competent TB40E and UV-TB40E elicited comparable CD4^+^ T-cell proliferative responses, we concluded that direct presentation of exogenous antigens is constitutively active in Mφ and is not enhanced by inflammatory cytokines released during direct infection. On the other hand, our data indicate that *de novo* synthesis of viral proteins is a prerequisite for optimal MHC class I mediated antigen presentation to CD8^+^ T cells.

An important consequence of the presence of MHC class I and II molecules on the surface of an infected cell is the capacity to present viral antigens to specific T cells and to induce T-cell activation and proliferation. At 1 as well as 3 dpi, CD4^+^ and CD8^+^ T cells vigorously proliferated in response to TB40E or ΔUS2–11-infected Mφ. Similarly to the HCMV-specific T cells present in healthy HCMV-seropositive subjects, the T lymphocytes responding to HCMV-infected Mφ had an effector memory phenotype, were activated and produced IFN-γ. Notably, HCMV-infected Mφ induced IFN-γ production in an average of 21% of the total CD4^+^ and 19% of the total CD8^+^ T cells, respectively; these percentages are much higher than those previously reported in co-culture studies performed with HCMV-infected DC (21, 22, 83, 84) and imply a crucial role for Mφ in skewing a T helper 1 response during HCMV infection. Even though IFN-γ is considered the best parameter for evaluation of HCMV-activated T cells (85), other cytokines such as TNF-α, IL-4, IL-6, IL-17, TGF-β, and IL-10 should be considered for a full characterization of the HCMV-dependent T-cell activation. Thus, we cannot exclude that a proportion of the T cells stimulated by HCMV-infected Mφ differentiate into other T-cell subsets. Finally, as important parameter reflecting the antiviral functionality of T cells, we measured the levels of the lytic granule marker CD107a on CD8^+^ T-cells stimulated by HCMV-infected Mφ. High percentages of CD8^+^ T cells exposed the lysosome-associated membrane glycoprotein CD107a on their cell surface upon stimulation with HCMV-infected M1- or M2-Mφ, suggesting efficient release of granzymes and perforin (49).

In HCMV-seropositive donors, we did not observe differences between the T-cell responses elicited by Mφ infected with the wild type TB40E or the ΔUS2-11 virus lacking the four immune evasive genes; TB40-infected Mφ and ΔUS2–11-infected Mφ induced comparable CD4^+^ and CD8^+^ cell proliferation as well as increased expression of the lytic granule marker CD107a on CD8^+^ cells. This is in contrast with findings in the rhesus macaques animal model where the MHC class I inhibitory function of US2–11 gene products has been shown to be crucial for CMV secondary infection and viral evasion from CD8^+^ T cells (86). The contradictory findings in these studies may be explained by the fact that results obtained by *in vivo* RhCMV superinfection studies performed in primates cannot be directly compared with observations derived from *ex vivo* infection of short-term cell cultures with blood cells obtained from HCMV-positive donors.

Altogether, our data reveal that HCMV-infected M1- and M2-Mφ are capable to bypass the inhibitory mechanisms ascribed so far to the immune evasive gene US2, US3, US6, and US11 and to specifically induce potent proliferation of memory CD4^+^ and CD8^+^ T cells. Furthermore, T cells that are stimulated by HCMV-infected Mφ are functionally active, as they over-express CD69, produce IFN-γ and express high levels of the lytic granule marker CD107a. Since Mφ are abundant, long living cells, and a site of HCMV reactivation, we suggest that these cells may account for the generation and maintenance of the large HCMV-specific T-cell pools present in the human host.

## Author Contributions

CL, SV, and GF conceived and designed the experiments. CL, CS, GF, and JM performed the experiments. CL, CS, SV, WB, TM, and GF analyzed and critically discussed the data. SV and GF wrote the manuscript.

## Conflict of Interest Statement

The authors declare that the research was conducted in the absence of any commercial or financial relationships that could be construed as a potential conflict of interest.

## Supplementary Material

Figure S1 At 3 dpi, while M1-Mφ are completely resistant to MHC class I and class II downregulation, a slight MHC downregulation takes place in M2-Mφ. Human foreskin fibroblasts (HFF), M1- and M2-Mφ were left untreated (mock) or infected with an MOI 5 of the wild type TB40E, the mutant ΔUS2–11 or the fibroblast-adapted HCMV strain AD169. **(A)** At 3 dpi, viable cells were labeled with anti-MHC-I, anti-MHC-II or isotypic control antibodies and examined by flow cytometry. Bars represent mean values ± SEM obtained from two HFF and three Mφ experiments. *p* calculated with Student’s two-sample equal variance *t*-test, with a two-tailed distribution. At 3 dpi, **(B)** M1- and **(C)** M2-Mφ cells were firstly incubated with PE-labeled anti-MHC-I, anti-MHC-II or isotypic control antibodies, then fixed, permeabilized, and incubated with the Alexa488-labelled anti-IE1-2. Bars represent mean fluorescence intensity values ± SEM obtained with cells from five different blood donors. *p* calculated with Student’s two-sample equal variance *t*-test, with a two-tailed distribution.
