# Supplementary figures and images for: Human Macrophages Escape Inhibition of Major Histocompatibility Complex-Dependent Antigen Presentation by Cytomegalovirus and Drive Proliferation and Activation of Memory CD4+ and CD8+ T Cells
Source: Front Immunol. 2018 May 25;9:1129. doi: 10.3389/fimmu.2018.01129 (PMC5981096; doi:10.3389/fimmu.2018.01129)

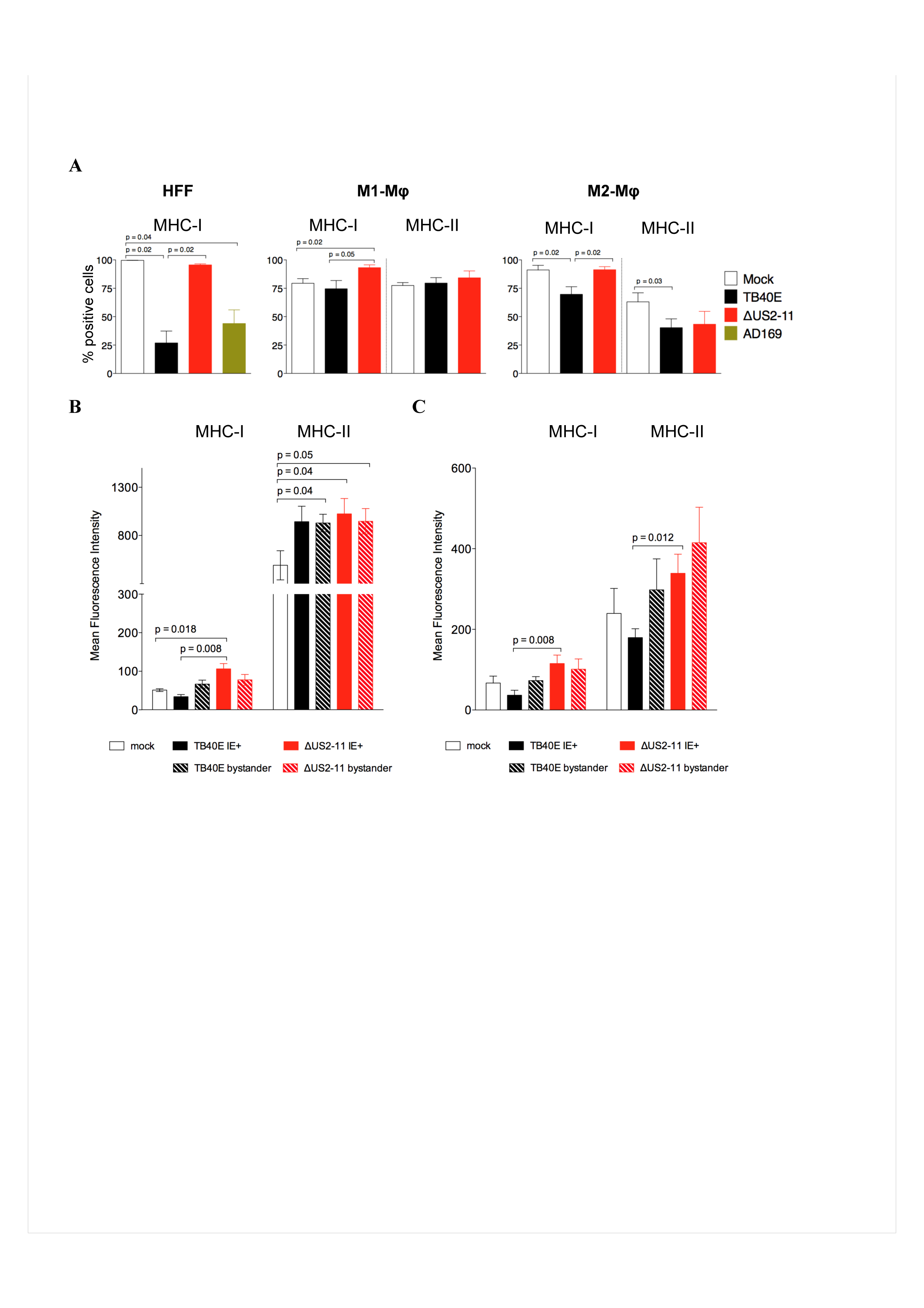

Supplement: Figure S1 — At 3 dpi, while M1-Mφ are completely resistant to MHC class I and class II downregulation, a slight MHC downregulation takes place in M2-Mφ. Human foreskin fibroblasts (HFF), M1- and M2-Mφ were left untreated (mock) or infected with an MOI 5 of the wild type TB40E, the mutant ΔUS2–11 or the fibroblast-adapted HCMV strain AD169. (A) At 3 dpi, viable cells were labeled with anti-MHC-I, anti-MHC-II or isotypic control antibodies and examined by flow cytometry. Bars represent mean values ± SEM obtained from two HFF and three Mφ experiments. p calculated with Student’s two-sample equal variance t-test, with a two-tailed distribution. At 3 dpi, (B) M1- and (C) M2-Mφ cells were firstly incubated with PE-labeled anti-MHC-I, anti-MHC-II or isotypic control antibodies, then fixed, permeabilized, and incubated with the Alexa488-labelled anti-IE1-2. Bars represent mean fluorescence intensity values ± SEM obtained with cells from five different blood donors. p calculated with Student’s two-sample equal variance t-test, with a two-tailed distribution. [file image_1.tif]
